# Methylferulate from *Tamarix aucheriana* inhibits growth and enhances chemosensitivity of human colorectal cancer cells: possible mechanism of action

**DOI:** 10.1186/s12906-016-1358-8

**Published:** 2016-10-01

**Authors:** Mohamed Salah I. Abaza, Mohammad Afzal, Raja’a J. Al-Attiyah, Radhika Guleri

**Affiliations:** 1 Molecular Biology Program, Department of Biological Sciences, Faculty of Science, P. O. Box 5969, Safat, 13060 Kuwait; 2 Biochemistry Program, Department of Biological Sciences, Faculty of Science, P. O. Box 5969, Safat, 13060 Kuwait; 3 Microbiology and Immunology Department, Faculty of Medicine, Kuwait University, Kuwait City, Kuwait

**Keywords:** *Tamarix aucheriana*, Methylferulate, Colorectal cancer, Apoptosis, Molecular mechanisms, Chemosensitization

## Abstract

**Background:**

Natural products are valuable sources for anticancer agents. In the present study, methylferulate (MF) was identified for the first time from *Tamarix aucheriana*. Spectral data were used for identification of MF. The potential of MF to control cell growth, cell cycle, apoptosis, generation of reactive oxygen species (ROS), cancer cell invasion, nuclear factor kappa B (NFkB) DNA-binding activity and proteasomal activities, as well as the enhancement of chemosensitivity in human colorectal cancer cells, were evaluated. The possible molecular mechanism of MF’s therapeutic efficacy was also assessed.

**Methods:**

Column chromatography and spectral data were used for isolation and identification of MF. MTT, immunofluorescence, flow cytometry, in vitro invasion, fluoremetry, EIA and Real time qPCR were used to measure antiproliferative, chemo-sensitizing effects and other biochemical parameters.

**Results:**

MF showed a dose-dependent anti-proliferative effect on colorectal cancer cells (IC_50_ = 1.73 – 1.9 mM) with a nonsignificant cytotoxicity toward normal human fibroblast. Colony formation inhibition (*P* ≤ 0.001, 0.0001) confirmed the growth inhibition by MF. MF arrested cell cycle progression in the S and G2/M phases; induced apoptosis and ROS generation; and inhibited NF-kB DNA-binding activity, proteasomal activities and cell invasion in colorectal cancer cells. MF up-regulated cyclin-dependent kinase inhibitors (p19 ^INK4D^, p21^WAF1/CIP1^, p27^KIP1^), pro-apoptotic gene expression (Bax, Bad, Apaf1, Bid, Bim, Smac) and caspases (caspase 2, 3, 6, 7, 8, 9). Moreover, MF down-regulated cyclin-dependent kinases (Cdk1, Cdk2) and anti-apoptotic gene expression (c-IAP-1, c-IAP-2, Bcl2,FLIP). In addition, MF differentially potentiated the sensitivity of colorectal cancer cells to standard chemotherapeutic drugs.

**Conclusion:**

MF showed a multifaceted anti-proliferative and chemosensitizing effects. These results suggest the chemotherapeutic and co-adjuvant potential of MF.

## Background

Cancer is a major health problem in both developed and developing countries. Worldwide, it is the second leading cause of death [1], with nearly 14 million new cases and 8.2 million cancer-related deaths in 2012 [2]. Colorectal cancer (CRC) is one of the most common forms of lower gastrointestinal cancer and around 75 % cases of CRC can be attributed to sporadic disease influenced by environmental factors and dietary lifestyle. The remaining 25 % of cases have a family history of CRC associated with hereditary or shared exposure among family members [3].

Phytotherapy has been used since antiquity. This type of therapy provides an extensive reservoir of structurally diverse natural products with distinct activities [4]. The predominant role of phytochemicals in health care is supported by a World Health Organization report indicating that 80 % of the global population uses herbal medicine for its primary health care. Today, 50 % of all drugs in clinical use and 74 % of the most important drugs are derived from phytochemicals [5]. Currently, more than 60 % of commercially available anticancer drugs are derived from natural sources, including plants, marine organisms and microorganisms [6].

To date, more than 3000 diverse plant species have been used in the treatment of cancer [7]. Four reasons may account for the continued interest in the investigation of phytochemicals for anticancer drug development. First, plants often produce complex bioactive molecules that exceed the current capacity of synthetic organic chemistry [8]. Second, natural anticancer compounds perfectly fit into a mechanism-based approach. Convincing evidence shows that phytochemicals can inhibit cancer by disrupting multiple mechanisms that are central to cancer progression [9]. Third, there are 2.5–5.0 million known terrestrial plants species and many more under the sea, but only less than 10 % of these species have been analyzed for their major constituents [10]. Today techniques have become available that can separate and identify minor components that may be equally important as major phytochemicals. Thus, the identification of novel structures and understanding their molecular mechanism can greatly contribute to specific strategies for the development of successful chemotherapies. Fourth, natural compounds such as taxol, a minor component of *Taxus brevifolia,* are being successfully used in cancer treatment [11].

Although there are new approaches to drug discovery, such as combinatorial chemistry and computer-based molecular modeling design, natural bioactive compounds still play, and will continue to play, a leading role in the discovery of effective drugs for the treatment of cancers [4, 12].

Compared with conventional anticancer drugs, plant-derived polyphenols have an extra margin of safety because they show marginal toxicity even at relatively high concentrations [13]. Unlike synthetic drugs that act as mono-target molecules, phytochemicals are multi-target molecules that regulate cancer growth and progression [14]. Although many studies have described the role of polyphenols, less attention has focused on simple phenolic acids in cancer prevention and antigenotoxicity [15]. Some *Tamaraix* (Tamaricaceae) species are widely used in traditional medicine in Asia and Africa [16]. For example, boiled leaves and young branches of *Tamarix* are used for the treating spleen edema. Mixed with ginger, the extract is used for uterus infections, prolonged and difficult labor, diverse sores and wounds [16]. Its tannins are used for the treatment of leukoderma, spleen problem, eye diseases, rheumatism, jaundice and hepatic disorders [17, 18]. The aim of the present study was to isolate and identify the potential chemotherapeutic/preventive constituents of *Tamarix aucheriana* using bioactivity-guided fractionation. The potential of MF to control cell growth, cell cycle, apoptosis, ROS generation, cancer cell invasion, NF-kB DNA-binding activity, and various proteolytic activities of proteasome, as well as the augmentation of the sensitivity to standard chemotherapeutic drugs of human colorectal cancer cells, was evaluated. The molecular mechanism of MF’s therapeutic value was also investigated.

## Methods

### Cell lines and chemicals

Human colorectal cancer cell lines (SW1116 and SW837) and normal human fibroblasts (CRL1554) were obtained from the American Type Culture Collection, ATCC (VA, USA). Leibovitz’s L-15 and EMEM (Eagle Minimum Essential Medium), trypsin, penicillin/streptomycin solution and fetal bovine serum (FBS) were obtained from Mediatech Inc. (Herndon, VA, USA). Primers, Taqman probes and all of the reagents for RT-PCR and real-time quantitative PCR (qPCR) were obtained from Applied Biosystems (Carlsbad, CA). The DNA-prep kit was obtained from Beckman & Coulter (Kendall, FL), and an Annexin V-FITC apoptosis detection kit was obtained from Hoffmann-La Roche Inc. (Nutley, NJ, USA). NFkB (p65) transcription factor assay kit was obtained from Cayman Chemical (Ann Arbor, MI, USA) and nuclear/cytosol fractionation kit was purchased from BioVision Inc. (Milipitas, CA, USA). Organic solvents of high-performance liquid chromatography (HPLC) grade were purchased from Fisher Scientific (Atlanta, GA, USA). Drugs, standard ferulic acid (FA) and other chemicals were obtained from Sigma-Aldrich Chemicals (St Louis, MO, USA).

### Plant material

*Tamarix aucheriana* (Decne.) Baum (Tamaricaceae) was collected during spring 2007 from Kuwait desert. Aerial parts of the plant, including stems, leaves, flowers and /or fruits, were collected, shade-dried and separately powdered. The plant was identified by the Herbarium Curator at Kuwait University, and a voucher specimen KTM 5461 was deposited in the university herbarium.

### Isolation and purification of MF from *Tamarix aucheriana*

The overground part of the powdered plant sample (100 g) was Soxhlet extracted with petroleum ether (40–60 °C), followed by methanol extraction. The methanolic extract (4.0 % yields), obtained after removal of the organic solvent under reduced pressure, was fractionated on a silica gel column (300–400 mesh, Silicycle, Cubec, Canada) packed in toluene. The column was eluted with toluene, chloroform followed by an increasing percentage of methanol in chloroform (30:70 v/v). Seven fractions (F1-F7, 50 mL each) were collected. Fraction 2 was a mixture of five components, as indicated by thin layer chromatographic (TLC) analyses, in a toluene: acetic acid: H_2_O (10:15:1, v/v) solvent system as a mobile phase. A component with an R_F_ value 0.35 was the major component of this fraction, and it was further purified by silica gel chromatography. The major compound thus purified showed a single spot in various TLC solvent systems, and for its identification, UV, IR, MS, H^1^-NMR and C^13^-NMR spectral data were collected.

### Cell culture

Human colorectal cancer cell lines (SW1116, passage # 41 and SW837, passage # 49) were cultivated in Leibovitz’s L15 medium (90 %) and fetal bovine serum (10 %). L15 medium was used with a free gas exchange with air. The standard sodium bicarbonate/CO_2_ buffering system was replaced by a combination of phosphate buffer, free-base amino acid, higher level of sodium pyruvate and galactose. A CO_2_ and air mixture was detrimental to the cells when used with this medium for cultivation. If cells in L-15 were incubated with CO_2_, the medium could quickly turn acidic and likely kill the culture. Normal human fibroblasts (CRL1554) were cultivated in EMEM (90 %) and fetal bovine serum (10 %).

### Anti-proliferative effect of MF

Cell viability was measured using the MTT assay, which is based on the conversion of 3-(4,5-dimethylthiazol-2-yl)-2,5-diphenyltetrazolium bromide to formazan crystals by mitochondrial dehydrogenases [19]. Briefly, colorectal cancer cell lines (SW1116 and SW837) and normal human fibroblasts (CRL1554) were seeded (27 × 10^3^ cells/well) onto flat-bottom 96-well culture plates at 37 °C for 18 h in a CO_2_ or non-CO_2_ incubator depending on the nature of the cell lines. Cells were incubated for 24 h in a culture medium containing an increasing concentration of MF (0–2.2 mM). DMSO (0.1 %) was used as a vehicle control. After completion of the treatment period, the cell supernatant was discarded and 100 μl/well of (5 mg/ml) MTT reagent was added and the plate was incubated at 37 °C for 4 h. MTT solution was aspirated, and the formazan crystals thus formed were dissolved in 200 μl/well of a DMSO: ethanol (1:1 v/v) mixture and left at ambient temperature for 20 min. Changes in the absorbance were monitored at 540 and 650 nm in an ELISA reader (Labsystems, Finland). Data were calculated as the percentage of inhibition by the following equation: % Inhibition = [1- (OD_t_/OD_ut_) × 100]. OD_t_ and OD_ut_ indicate the optical density of cell lines incubated with MF and vehicle control, respectively. The cytotoxic effect of MF on cell lines was expressed as the IC_50_ value (the drug concentration reducing the absorbance of treated cells by 50 % with respect to that of untreated cells). All experiments were carried out in triplicate.

### Morphological examination

Morphological study was carried out to observe morphological changes in dead cells. For observing morphological changes, SW1116 and SW837 human colorectal cancer cells were plated (2.5 × 10^5^ cells/ml) into a 24-well plate in a non-CO_2_ incubator for 18 h. Cells were treated with MF (1.5 mM) for 24 h and observed under an inverted microscope (Carl Zeiss MicroImaging GmbH, Gottingen, Germany) (×200). The untreated cells served as a negative control.

### Colony formation assay

SW1116 and SW837 cells were plated (2.5 × 10^5^ cells/ml) for 18 h in a non-CO_2_ incubator. Cells were left untreated or treated with MF (1.5 mM) and incubated at 37 °C for 24 h. The cells were trypsinized, counted, and plated into a six-well plate at 500 cells/ml and incubated at 37 °C in a non-CO_2_ incubator for 10–14 d; while the growth medium was replaced every two days. Cells were fixed in 100 % methanol for 30 min at room temperature and stained with 0.1 % crystal violet for 1 h. The stained colonies were counted and compared with the control cells [20].

### Cell cycle analysis

Flow cytometry was used to monitor the disruption in cell cycle phases (Go/G1, S and G2/M) by measuring the DNA content of the nuclei labeled with propidium iodide (PI), as previously described [20]. Briefly, SW1116 and SW837 cells were plated (2.5 × 10^5^ cells/ml) into 24-well plates and incubated at 37 °C in a non-CO_2_ incubator. Cells were treated with MF (1.5 mM) for 24 h starting 18 h after seeding the cells in culture. Untreated and MF-treated cells were collected by trypsinization, washed with cold PBS and counted. Cells were processed using a DNA-prep kit (Beckman & Coulter, FL, USA) and a DNA-Prep EPICS workstation (Beckman & Coulter). During this process, cells were treated with a cell-membrane permeabilizing agent followed by a treatment with PI and RNAase followed by incubation at room temperature for 15 min before analysis by flow cytometry (FC500, Beckman & Coulter). The percentage of cells in different cell cycle phases was calculated using the Phoenix statistical software package (Phoenix Flow System, San Diego, CA).

### Analysis of apoptosis

The FITC-coupled annexin V detection kit from Roche was used to monitor apoptosis induction. Briefly, cancer cell lines SW1116 and SW837 were plated (2.5 × 10^5^ cells/ml) into a 24-well plate and incubated at 37 °C for 18 h in a non-CO_2_ incubator. Cells were treated with MF (1.5 mM) for 24 h. Cells from the control and treatment groups were resuspended in a 100 μl staining solution containing annexin-V fluorescein and propidium iodide. After incubation at room temperature for 15 min, the cells were analyzed by flow cytometry. Annexin V binds cells that express phosphatidylserine on the outer layer of the cell membrane and propidium iodide stains the cellular DNA with a compromised cell membrane. This approach allowed the differentiation of live cells (annexin V― PI―) from early apoptotic cells (annexin V+ PI―), late apoptotic cells (annexin V+ PI+) and necrotic cells (annexin V―, PI+).

### Assessment of ROS generation

To screen the production of ROS, human colorectal cancer cells (2.5 × 10^5^ cells/well) were seeded in a 24-well plate for 18 h. Cells were treated with MF (1.0 mM) for 24 h and detached by trypsin-EDTA. Subsequently the cells were washed with PBS, followed by a treatment with 20 μM dichlorofluorescein diacetate (DCF-DA) for 30 min, in the dark, at ambient temperature. The generation of intracellular ROS was visualized using an immunofluorescent microscope (Carl Zeiss MicroImaging GmbH, Gottingen, Germany). Changes in the fluorescence intensity relative to the untreated group were interpreted as an increase/decrease in the generation of intracellular ROS. Cell images were processed with ImageJ software [21]. ImageJ allows calculating mean grey value in outlined areas. Consequently, selected fluorescent cells integrated density (IntDen) could be obtained by multiplying measured grey value (MGV) to outlined cell area. With background mean grey value (BMGV), a Correlated Total Cell Fluorescence (CTCF) could be calculated by using the following equation:

$$ \mathrm{CTCF}=\mathrm{IntDen}-\left(\mathrm{Area}\;\mathrm{of}\;\mathrm{selected}\;\mathrm{cells}\times \mathrm{BMGV}\right). $$

### MF inhibits colorectal cancer cell invasion in vitro

In vitro inhibition of colorectal cancer cell invasion after treatment with MF was examined by using Chemicon’s cell invasion assay kit (Cayman Chemical, Ann Arbor, MI). Dried extracellular matrix (ECM) was re-hydrated by adding 300 μl of warm serum-free medium to the interior of the inserts, at room temperature for 1–2 h. Later, the medium was carefully removed from the inserts, and 500 μl of the medium containing 10 % FBS was added to the lower chamber. SW1116 and SW837 cells were plated (2.5 × 10^5^ cells/well) into 24-well plates, incubated at 37 °C in a non-CO_2_ incubator for 18 h and then treated with MF (1.5 mM) for 24 h, harvested and counted. Untreated and MF-treated cells were added to each insert (300 μl containing 0.5 - 1 × 10^6^ cells/ml in a serum-free medium) and incubated at 37° C for 24–72 h in a non-CO_2_. Cells were removed by inserting a cotton swab into the insert and gently applying firm pressure by moving the tip over the membrane surface. Dipping the insert in the staining solution for 20 min stained the invasive cells on the lower surface of the membrane. The inserts were rinsed with water, air-dried and counted by photographing the membrane through an inverted light microscope.

### Nuclear factor k-B DNA-binding activity

Cancer cell lines SW1116 and SW837 were plated (2.5 × 10^5^ cells/ml) into a 24-well plate in a non-CO_2_ incubator at 37 °C for 18 h, and treated with MF (1.5 mM) for 24 h. Nuclear extracts were purified by using a nuclear/cytosol fractionation kit (BioVision, Inc.). NF-kB (p65) activity was determined by Cayman’s NF-kB (p65) transcription factor assay. In this assay, a specific double-stranded DNA sequence containing the NF-kB response element was immobilized onto the bottom of the wells in a 96-well plate. The NF-kB (p65) of the nuclear extracts or positive control was detected by adding a specific primary antibody directed against NF-kB (p65). A second antibody conjugated to HRP was added to provide a sensitive colorimetric readout at 450 nm.

### Proteolytic activities of the proteasome

Cancer cell lines SW1116 and SW837 were plated (2.5 × 10^5^ cells/ml) into 24-well plates in a non-CO_2_ incubator at 37 °C for 18 h, and the cells were treated with MF (1.5 mM) for 24 h. Cytosolic fractions were prepared by using a nuclear/cytosolic fractionation kit (BioVision, Inc.). The cytosolic extracts (5 μg) of the untreated and MF-treated cancer cells were incubated with 20 μM fluorogenic substrates for various proteolytic activities of the proteasome, Suc-Leu-Leu-Val-Tyr-AMC (for proteasomal chymotrypsin-like activity), benzyloxycarbonyl(Z)-Leu-Leu-Glu-AMC (for proteasomal PGPH activity) and Z-Gly-Gly-Arg-AMC (for proteasomal trypsin-like activity) at 37 °C for 90 min in 100 μl of assay buffer (20 mM, Tris–HCl, pH 8.0). The reaction mixture was diluted to 200 μl with the assay buffer, hydrolyzed 7-amido-4-methyl-coumarin (AMC) was then measured using a VersaFluor™ fluorometer with an excitation wavelength of 360 nm and an emission 460 nm (Bio-Rad).

### mRNA level of apoptosis and cell-cycle-regulatory genes assessment

Expression of cell cycle and apoptosis regulatory genes were measured in control and MF-treated cells by real-time PCR [20]. All manipulations were carried out using an Applied Biosystems assay. The target and number of cell cycle regulatory genes were as follows: Cdk1 (Hs00364293_m1), Cdk2 (Hs00608082_m1), p19^INK4D^ (Hs00176481_m1), p21^WAF1/CIP1^ (Hs00355782_m1) and p27^KIP1^ (Hs00197366_m1). The targets and their numbers for pro-apoptotic, anti-apoptotic, and caspase genes were as follows: Bad (Hs188930_m1), Bax (Hs00180269_m1), Bid (Hs00609632_m1), Bim (Hs00375807_m1), Apaf1 (Hs00559441_m1) and Smac (Hs00219876_m1); cIAP-1 (Hs0023691_m1), c-IAP-2 (Hs00985029_m1), Bcl2 (Hs00608023) and FLIP (Hs00354474_m1); casp2 (Hs00154242_m1), casp3 (Hs00234387_m1), casp6 (Hs00154250_m1), casp7 (Hs00169152_m1), casp8 (Hs01018151_m1) and casp9 (Hs00154260_m1); and GAPDH. The latter was used as an endogenous control to normalize the expression values for each sample. For the comparative Ct method, we performed a two-step RT-PCR using cDNA and carried out real-time quantitation using the target gene expression assays and Taqman universal master mix. SW837 human colorectal cancer cells were plated (2.5 × 10^5^ cells/ml) into 24-well plates in a non-CO_2_ incubator at 37 °C for 18 h. Cells were treated with MF (1.5 mM) for 24 h. mRNA was extracted using nucleospin an RNAII ready-to-use system (MACHEREY-NAGEL). For the RT reaction, 200 ng/μl of mRNA was used. First, DNA was eliminated by DNase-I treatment for 20 min at 25 °C, followed by heat inactivation at 65 °C for 10 min. cDNA synthesis was performed using a high-capacity cDNA reverse transcription kit according to the manufacturer’s instructions. For each sample, 2.5 μl of cDNA and 12.5 μl of Taqman universal master mix (2×) were used, and the volume was adjusted to 25 μl with nuclease-free water in a 96-well reaction optical plate. Real-time RT-PCR was performed on an ABI 7000 SDS system using ABI Prism’s SDS collection software version 1.1. Real-time RT-PCR conditions followed the Taqman universal master mix manufacturer’s protocol: step 1 at 95 °C for 10 min; step 2 at 94 °C for 15 s; and step 3, at 60 °C for 1 min. The amount of target, normalized to an endogenous reference and relative to a calibrator (untreated), was given by 2^-∆∆Ct^. The log comparative Ct was presented graphically. 2^-∆∆Ct^ gave linear form representing the factor change in the gene expression.

### MF potentiates standard anticancer drugs

The potential of MF to sensitize human colorectal cancer cells to standard chemotherapeutic drugs was investigated as previously described [20]. SW1116 and SW837 Cancer cells were plated (27 × 10^3^ cells/well) into a 96-well plate at 37 °C in a non-CO_2_ incubator for 18 h. After starting the culture, the cells were treated for 24 h with various concentrations of camptothecin (CPT, 128× 10^−11^ – 1.0 × 10^−4^ M), 5-fluorouracil (5FU, 89.6× 10^−10^– 0.7× 10^−3^ M), doxorubicin (DOX, 110 × 10^−12^ – 0.86× 10^−5^ M), oxaliplatin (OXP, 7.6× 10^−11^ – 0.06× 10^−4^ M), taxol (TAX, 94 × 10^−11^ – 1.47 × 10^−4^ M), vinblastine (VBL, 3.84× 10^−11^ – 0.03× 10^−4^ M), vincristine (VCR, 3.84× 10^−11^ – 0.03× 10^−4^ M), etoposide (ETP, 5.12× 10^−10^ – 0.04 × 10^−3^ M), ellipticine (ELP, 2.56× 10^−10^ – 0.02× 10^−3^ M), amsacrine (AMS, 1.28× 10^−10^ – 0.01× 10^−3^ M), homoharrigtonine (HHG, 2.56× 10^−12^ – 0.2 × 10^−5^ M), and aphidicolin (APD, 38.42 × 10^−12^ – 0.3× 10^−5^ M). The drug was removed and the cells were washed with Hankes balanced salt solution (HBSS) and treated with MF (1.5 mM) for 24 h, and cell growth was monitored by MTT assay.

### Statistical analyses

Data were reported as the means ± SEM. Significant differences between experimental groups were assessed by one-way ANOVA followed by Posthoc and LSD with SPSS (version 22.0), with significance level set at *P* < 0.05.

## Results

### Identification of MF from *T. aucheriana*

**Fig. 1:**
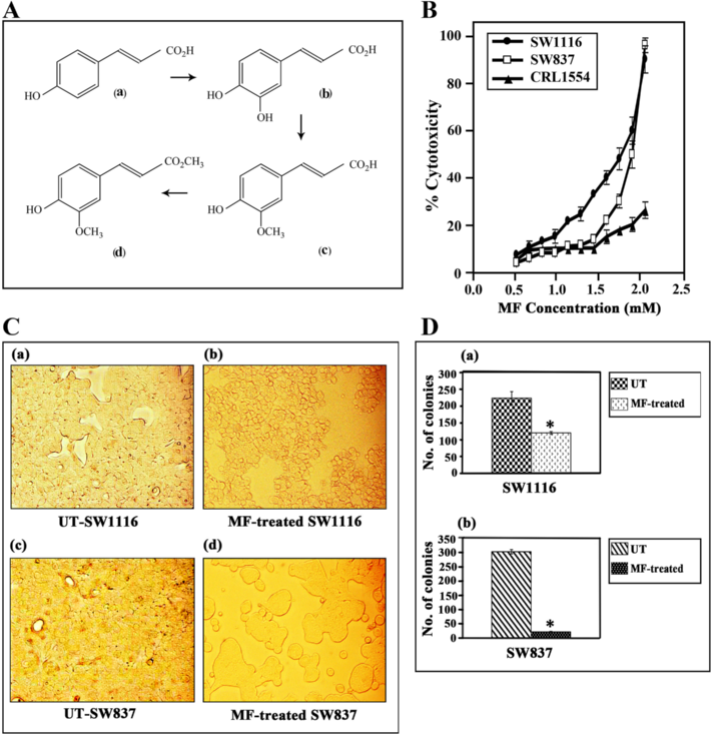
Cytotoxicity of MF on human colorectal cancer cell lines and normal human fibroblast cells. **A** Biosynthesis of hydroxycinnamic acids in plants: (*a*) *p*-coumaric acid, (*b*) caffeic acid, (*c*) ferulic acid, (*d*) methylferulate. **B** Growth inhibition of colorectal cancer cell lines SW1116 and SW837 as well as normal human fibroblast cells of the line CRL1554. **C** Morphological changes in colorectal cancer cells treated with MF. **D** Colony formation by untreated and MF-treated cancer cell lines. Data are reported as the means ± SE of three independent experiments, *P* ≤ 0.05 compared with untreated

UV, IR, MS, H^1^-NMR and C^13^-NMR spectral data confirmed the identity of the compound as 4-hydroxy-3-methoxymethylcinnamate (ferulic acid methyl ester/methylferulate). It gave a positive ferric chloride test, indicating its phenolic nature. The accurate mass measurement (208.057802 Da) confirmed its molecular formula (C_12_H_14_O_4_), which agrees with the standard 4-hydroxy-3-methoxy-methylcinnamate (Fig. 1A). Standard methylferulate was obtained by esterification of ferulic acid in dry acidified methanol. All spectral data (Table 1) for the synthetic product agreed with the natural product, thus identifying the isolated natural material. The purity of the synthetic and natural methylferulate was > 99 %.

**Table 1:** Spectral data for MF identification

- UV absorption showed at 244 (1.027); 295 (1.507); 319 (1.675)	
- IR absorption bands showed at cm^−1^ 3536.8 (Ph-OH); 2917.77, 2848.33 (>CH stretching); 1701.87 (conjugated ester > C = O); 1614.47 (Ar-C = C); 1198.54, 1129.12 (doublet for ester function); 731.58 (trisubstituted Ph ring).	
-^1^HNMR (CDCl_3_), 600 MHz; ppm: 7.631, 7.604 (d, 8.5 Hz), 1H, (Ar-H); 7.155 (s), (Ar-H); 70.55, 7.042, (d, Hz 8.1 Hz), (Ar-H); 6.970, 6.856,d, 9.8Hz, (Ar-CH = CH-C = O-); 6.322, 6.296, d(9.2 Hz), (Ar-CH = CH-C = O-), 5.638,s, Ar-H; 3.946,s,3H, Ar-COOCH_3_; 3.808, s, 3H, Ar-OCH_3_.	
- ^13^CNMR: 167.73 (>C = O); 115.86 (Ar-CH = CH-COOCH_3_); 144.67, (Ar-CH = CH-COOCH_3_); 145.83, 148.49, 128.03. 121.85, 115.86, 112.95, 110.49 (Ar-C); 56.01, (−COOCH_3_); 55.62, (Ar-OCH_3_)	
- MS,EI: 208 (M+.); 193 (M-CH_3_); 177 (M—OCH_3_); 149 (177- > C = O); 133, 117, 89	

### Cell proliferation inhibition by MF

The cytotoxicity of MF at variable concentrations (0 – 2.2 mM) was monitored using the MTT assay. MF exerted a dose-dependent growth inhibition of human colorectal cancer cells, with IC_50_ values 1.73 mM and 1.9 mM for SW1116 and SW837, respectively (Fig. 1B). A 24-hour treatment of SW1116 and SW837 with MF (1.5 mM) resulted in gross morphological changes, as observed under the inverted microscope. The cellular morphology was rigorously distorted after treatment with MF, and some of the cells appeared round, whereas most cells appeared withered and arched on the culture surface (Fig. 1C). Significant inhibition of colony formation for both SW1116 (mean of colonies for UT = 233 ± 10 vs. 122 ± 2 for MF-treated cells, *P* ≤ 0.001) and SW837 (mean of colonies for UT = 305 ± 4 vs. 22 ± 1.0 for MF-treated cells, *P* ≤ 0.0001) was observed (Fig. 1D).

### MF induced cell cycle arrest

**Fig. 2:**
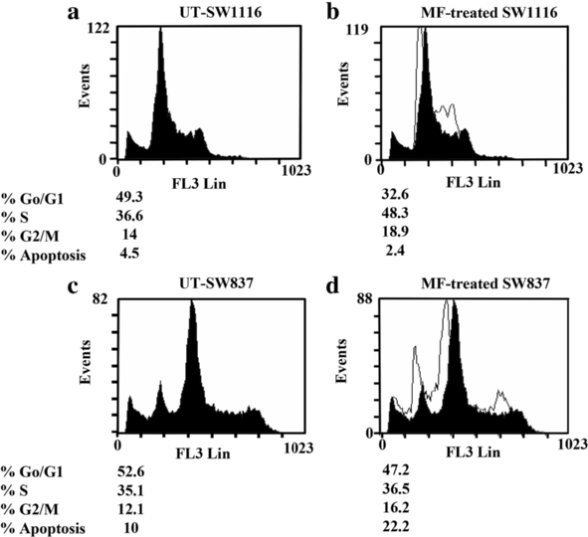
Flow cytometry of cell cycle phase distribution of human colorectal cancer cells treated with MF. Colorectal cancer cells SW1116 and SW837 were treated with MF (1.5 mM) for 24 h. Cell cycle proportions were determined by flow cytometry after staining with propidium iodide. At least three samples were analyzed and 20,000 events were scored for each sample. The vertical axis represents the relative number of events, and the horizontal axis represents fluorescence intensity. **a**, **b**: Untreated and MF-treated SW1116; **c**, **d**: Untreated and MF-treated SW837

Human colorectal cancer cells were treated with MF and harvested for flow cytometric analyses. SW1116 cells accumulated in the S-phase (48.3 % vs. 36.6 % for untreated UT) and G_2_/M-phase (18.9 % vs. 14 % for UT). This accumulation occurred at the expense of a conclusive decrease in the G_1_/G_o-_phase (32.6 % vs. 49.3 % for UT) (Fig. 2a, b). Similar results were obtained with SW837, which showed an increase in cell population in the S-phase (36.5 % vs. 35.1 % for UT) and G_2_/M-phase (16.2 % vs. 12.1 % with UT), with a corresponding decrease in the number of cells in G_1_/G_o-_ phase (47.2 % vs. 52.6 % for UT) (Fig. 2c, d). An increase in the percentage of sub-G_1_ implied an increase in the percentage of apoptotic cells.

### Apoptosis triggered by MF

**Fig. 3:**
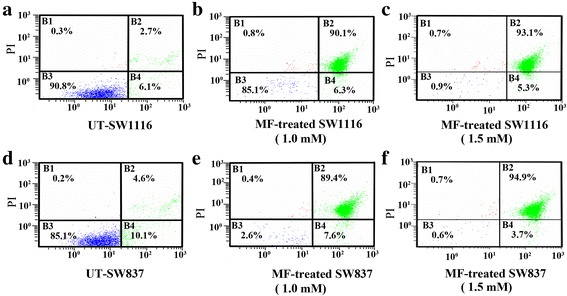
Induction of apoptosis in human colorectal cancer cells after treatment with MF by flow cytometric analysis. Colorectal cancer cell lines SW1116 and SW837 were treated with MF (1.0 and 1.5 mM) for 24 h. Cells were double stained with annexin V and FITC and analyzed by flow cytometry. *B1* Percentage of necrotic cells, *B2* percentage of late apoptotic cells, *B3* percentage of living cells, and *B4* percentage of early apoptotic cells. **a**, **b/c**: Untreated and MF-treated SW1116; **d**, **e/f**: Untreated and MF-treated SW837.

Annexin V binding to the cell surface was carried out in conjunction with PI staining [22]. Untreated SW1116 cells showed a very low level of apoptosis, with 6.1 % exhibiting early apoptosis, 2.7 % exhibiting late apoptosis and 0.3 % exhibiting necrosis (Fig. 3a). Among SW1116 cells treated with MF (1.0 mM) for 24 h, 6.3 % showed early apoptosis, 90.1 % showed late apoptosis, and 0.8 % showed necrosis (Fig. 3b). Meanwhile, SW1116 cells treated with MF (1.5 mM) for 24 h, 5.3 % showed early apoptosis, 93.1 % showed late apoptosis, and 0.7 % showed necrosis (Fig. 3c). On the other hand, untreated SW837 cells exhibited low level of apoptosis, with 10.1 % exhibiting early apoptosis, 4.6 % exhibiting late apoptosis and 0.2 % showed necrosis (Fig. 3d). Among SW837 cells treated with MF (1.0 mM) for 24 h, 7.6 % showed early apoptosis, 89.4 % showed late apoptosis and 0.4 % showed necrosis (Fig. 3e). Moreover, among SW837 cells treated with MF (1.5 mM) for 24 h, 3.7 % showed early apoptosis, 94.9 % showed late apoptosis and 0.7 % showed necrosis (Fig. 3f). The distinct increase in the percentage of sub-G_1_ indicated an increase in the percentage of apoptotic cells (Fig. 2).

### MF generates ROS

**Fig. 4:**
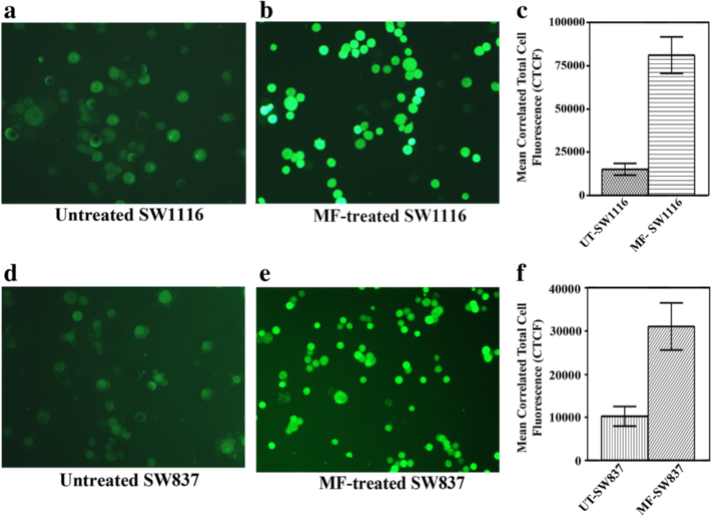
Induction of reactive oxygen species generation by MF. The generation of intracellular ROS was visualized using an immunofluorescent microscope (**a**, **b**, **d**, **e**). Cell images were processed by ImageJ software (**c**, **f**). *P* ≤ 0.05 compared with MF-treated. CTCF: Correlated Total Cell Fluorescence

ROS has implicated as second messengers in multiple signaling pathways that play an important role in apoptosis [23]. ROS generation by MF was evaluated by DCFH-DA, which is cleaved by the intracellular nonspecific esterase to form DCFH. Untreated colorectal cancer cells (control) showed very slight fluorescence (Fig. 4a, d). On the other hand, a marked increase in fluorescence intensity was observed in cancer cells treated with MF (Fig. 4b, e). Changes in the fluorescence intensity of MF-treated cells relative to that of untreated cells were inferred as an increase in the intracellular ROS. Cell images were processed with ImageJ software. CTCF corresponds to a relative unit that can be used to quantify cell fluorescence. The MF-treated SW1116 (*P* ≤ 0.0001) and MF-treated SW837 cells (*P* ≤ 0.008) appeared to have a much higher level of staining than untreated SW 1116 (Fig. 4c) and untreated SW837 (Fig. 4f).

### Inhibition of cancer cell invasion by MF

**Fig. 5:**
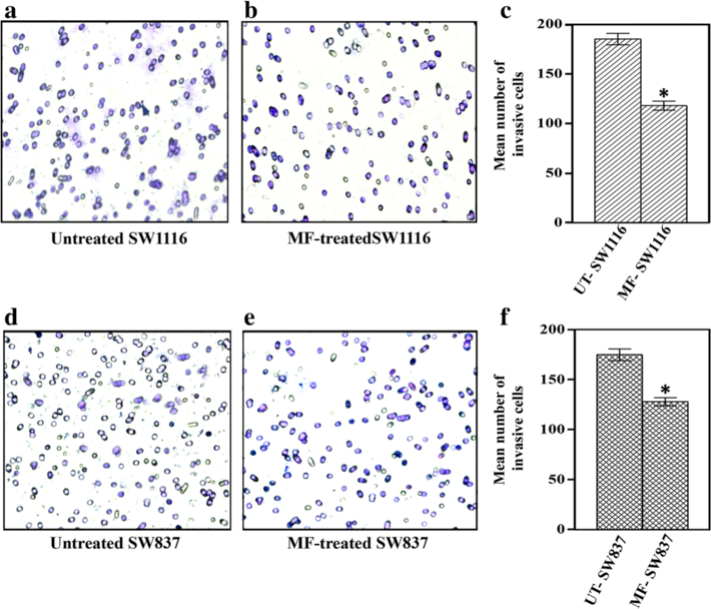
MF inhibits in vitro invasion of human colorectal cancer cells. In vitro invasion assay was performed by Chemicon’s cell invasion assay kit. *P* ≤ 0.05 compared with untreated. **a**, **b**, **c**: Untreated and MF-treated invasive SW1116 cells; **d**, **e**, **f**: Untreated and MF-treated invasive SW837 cells

The inhibition of cancer cell invasion by MF was examined by using Chemicon’s cell invasion assay. The number of both SW1116 (Fig. 5b, *P* ≤ 0.006) and SW837 (Fig. 5e, *P* ≤ 0.031) cells at the bottom of the polycarbonate membrane markedly decreased after treatment with MF (1.5 mM) (Fig. 5c, f) compared with the untreated control (Fig. 5a, d).

### MF Inhibits proteasome and NF-kB DNA-binding activities

**Fig. 6:**
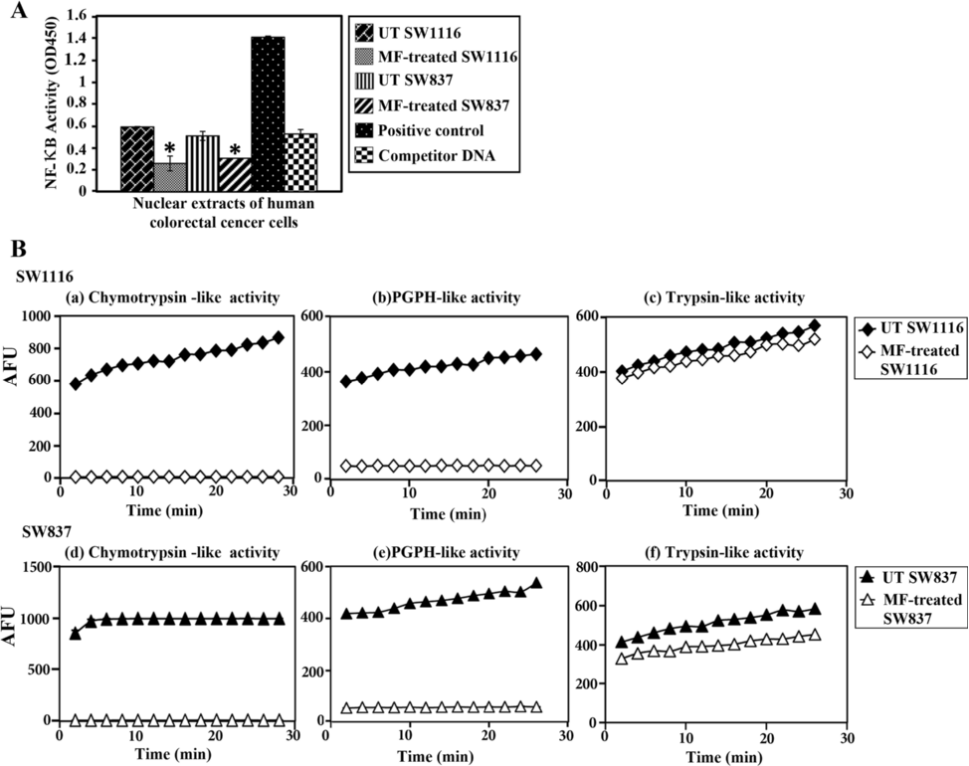
Inhibition of NFkB and proteasome activities by MF in human colorectal cancer cells. **A**: NFkB DNA binding activity was determined in nuclear extracts. **B**: Proteasomal activities were determined in cytosolic extracts as described in the Methods. *P* ≤ 0.05 compared with untreated

There was a significant decrease in the DNA-binding activity of NF-kB in SW1116 (*P* ≤ 0.004) and SW837 (*P* ≤ 0.022) cells treated with MF (1.5 mM) (Fig. 6A). The effect of MF (1.5 mM) on the various proteolytic activities of the ubiquitin-proteasome system was evaluated. MF significantly inhibited the chymotrypsin-like activity (*P* ≤ 0.0001) (Fig. 6Ba) and PGPH activity (*P* ≤ 0.0001) (Fig. 6Bb) but not the trypsin-like activity (*P* ≤ 0.447) (Fig. 6Bc) of the 26S proteasome in the cytosolic extract of SW1116. In addition, MF significantly inhibited the chymotrypsin-like (*P* ≤ 0.0001) (Fig. 6Bd) and PGPH (*P* ≤ 0.0001) (Fig. 6Be) activities, as well as marginally affected the trypsin-like activity (*P* ≤ 0.065) (Fig. 6Bf), of 26S proteasome in the cytosolic extract of SW837.

### Gene expression in colorectal cancer cells treated with MF

**Fig. 7:**
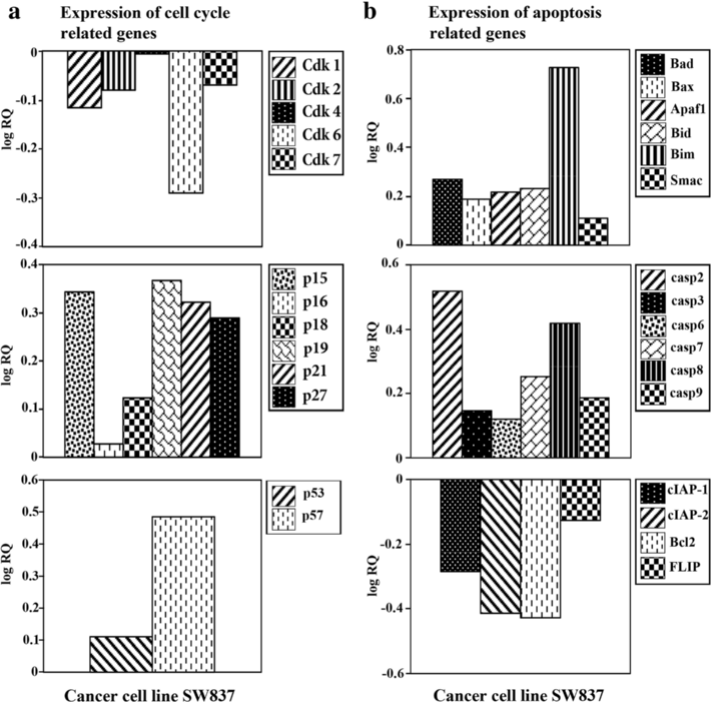
mRNA expression of the genes controlling cell cycle and apoptosis in cancer cells treated with MF. **a** Assessment of the mRNA expression of cell-cycle-regulatory genes. **b** Assessment of the mRNA expression of apoptosis-regulatory genes

MF differentially down-regulated the gene expression of Cdk1 and Cdk2 (Fig. 7a) and the anti-apoptotic genes, including cIAP-1, c-IAP-2, Bcl2, and FLIP (Fig. 7b). On the other hand, MF differentially up-regulated the gene expression of p19^INK4D^, p21^WAF1/CIP1^ and p27^KIP1^ (Fig. 7a) and the expression of the pro-apoptotic genes, including Bax, Bad, Bid, Bim, Apaf-1, Smac and the expression of caspases’genes, including caspase-2, 3, 6, 7, 8, and 9 (Fig. 7b).

### Enhancement of cytotoxicity of standard chemotherapeutic drugs by MF

**Fig. 8:**
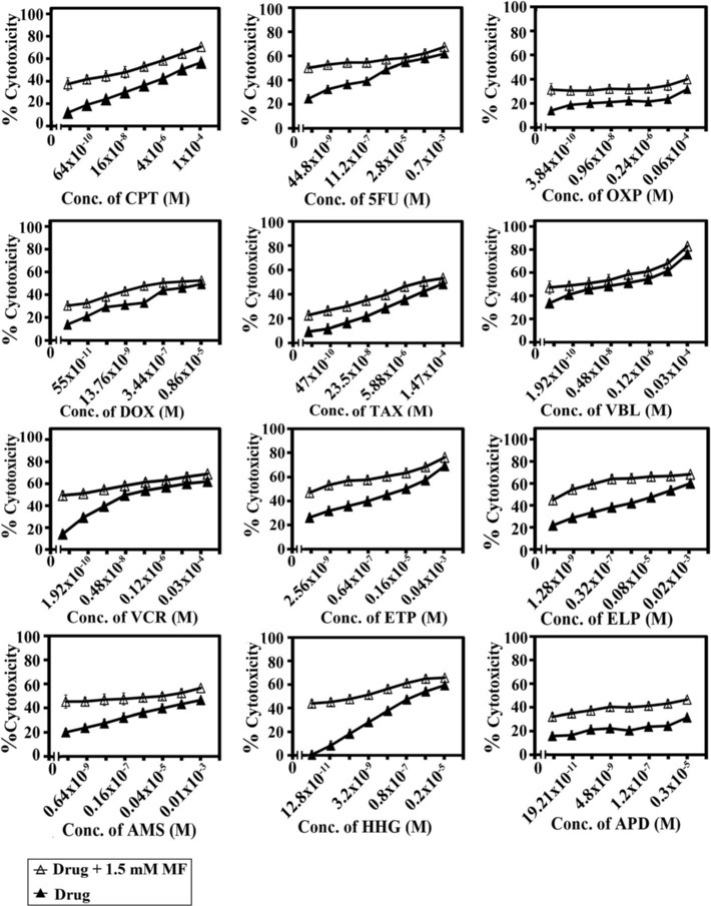
Enhancement of chemosensitivity of colorectal cancer cell line SW1116. Cell proliferation was monitored by MTT assay

**Fig. 9:**
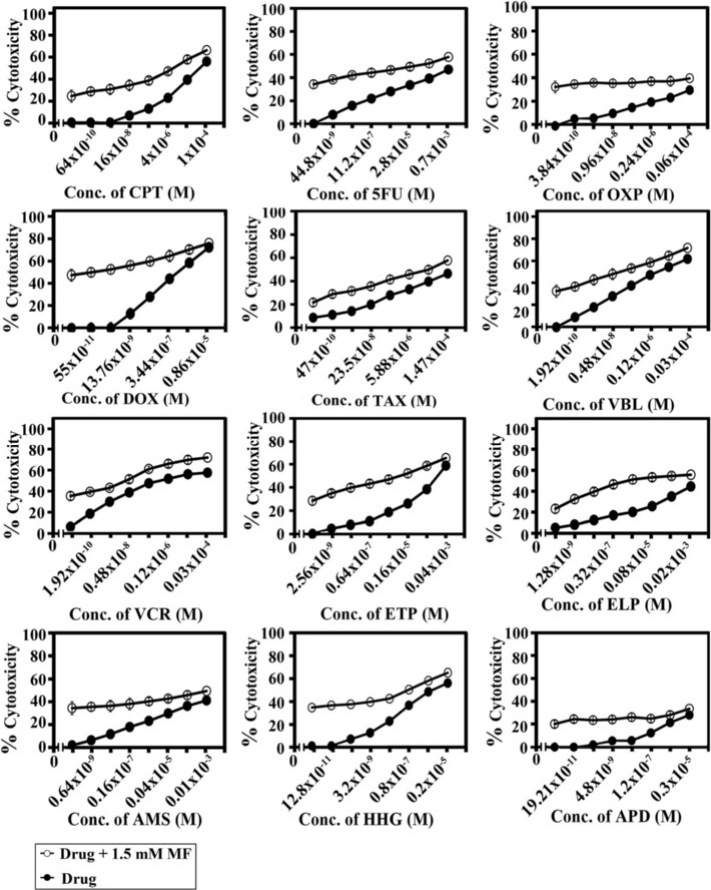
Enhancement of chemosensitivity of colorectal cancer cell line SW837. Cell proliferation was monitored by MTT assay

The potential of MF to sensitize human colorectal cancer cells to standard chemotherapeutic drugs was investigated. MF concentration (1.5 mM), used in the combination study, was based on the dose response inhibition study in Fig. 1B. MF concentration (1.5 mM) produced 10– 25 % cytotoxicity. The IC_50_ values and the sensitization ratios were used as measures of the ability of MF to potentiate the sensitivity of human colorectal cancer cell lines SW1116 (Fig. 8) and SW837 (Fig. 9) (Table 2) to standard chemotherapeutics with different mechanism of action. Our results demonstrated the differential sensitization of human colorectal cancer cell line SW1116 to standard chemotherapeutic drugs, with a marked increase in its sensitivity to CPT (Sensitization Ratio: SR = 95), 5FU (SR = 1051), DOX (SR = 125), VCR (SR = 254), ETP (SR = 2204), ELP (SR = 4615) and AMS (SR = 650). Moreover, differential sensitization to the tested chemotherapeutic drugs was observed with cancer cell line SW837, with a marked increase in its sensitivity to 5 FU (SR = 269) and ELP (SR = 625) (Table 2).

**Table 2:** IC_50_ values and sensitization ratio of conventional chemotherapeutic drugs and its combinations with MF towards human colorectal cancer cell lines SW1116 and SW837

Single and combined treatment with chemotherapeutic drugs and MF	SW1116		
	IC_50_ (M)^a^	Sensitization ratio^b^	*P* ^c^ values
CPT (128× 10^−11^- 1× 10^−4^ M)	2.0 × 10^−5^	-	
CPT (128× 10^−11^ - 1× 10^−4^ M) + MF (1.5 mM)	21 × 10^−8^	95.0	0.025
5FU (89.6× 10^−10^- 0.7× 10^−3^ M)	8.83 × 10^−6^	-	
5FU (89.6× 10^−10^- 0.7× 10^−3^ M) + MF (1.5 mM)	84 × 10^−10^	1051	0.025
DOX (110× 10^−12^ - 0.86× 10^−5^ M)	0.43 × 10^−4^	-	
DOX (110× 10^−12^ - 0.86× 10^−5^) + MF (1.5 mM)	3.44 × 10^−7^	125	0.081
TAX (94× 10^−11^- 1.47× 10^−4^ M)	1.47 × 10^−4^	-	
TAX (94× 10^−11^- 1.47× 10^−4^ M) + MF (1.5 mM)	5.72 × 10^−6^	26	0.183
VBL (3.84× 10^−11^ - 0.03× 10^−4^ M)	0.22 × 10^−8^	-	
VBL (3.84× 10^−11^ - 0.03× 10^−4^ M) + MF (1.5 mM)	3.52 × 10^−11^	62.55	0.326
VCR (3.84× 10^−11^ - 0.03× 10^−4^ M)	0.4 × 10^−8^	-	
VCR (3.84× 10^−11^ - 0.03× 10^−4^ M) + MF (1.5 mM)	3.15 × 10^−11^	254	0.079
ETP (5.12× 10^−10^ - 0.04× 10^−3^M)	1.19 × 10^−6^	-	
ETP (5.12× 10^−10^ - 0.04× 10^−3^M) + MF (1.5 mM)	5.4 × 10^−10^	2204	0.026
ELP (2.56× 10^−10^ - 0.02× 10^−3^M)	0.06 × 10^−4^	-	
ELP (2.56× 10^−10^ - 0.02× 10^−3^M) + MF (1.5 mM)	1.3 × 10^−9^	4615	0.001
AMS (1.28× 10^−10^- 0.01× 10^−3^M)	0.013 × 10^−3^	-	
AMS (1.28× 10^−10^- 0.01× 10^−3^M) + MF (1.5 mM)	0.2 × 10^−7^	650	0.001
HHG (25.6× 10^−12^ – 0.2× 10^−5^M)	2.0 × 10^−7^	-	
HHG (25.6× 10^−12^ – 0.2× 10^−5^M) + MF (1.5 mM)	3 × 10^−9^	67	0.010
Single and combined treatment with chemotherapeutic drugs and MF	SW837		
	IC_50_ (M)^a^	Sensitization ratio^b^	*P* ^c^ values
CPT (128× 10^−11^- 1× 10^−4^ M)	5 × 10^−5^	-	
CPT (128 × 10^−11^- 1× 10^−4^ M) + MF (1.5 mM)	4 × 10^−6^	12.5	0.012
5FU (89.6× 10^−10^- 0.7× 10^−3^ M)	0.7 × 10^−3^	-	
5FU (89.6× 10^−10^- 0.7× 10^−3^ M) + MF (1.5 mM)	2.6 × 10^−5^	269	0.001
DOX (110× 10^−12^ - 0.86× 10^−5^ M)	3.44 × 10^−6^	-	
DOX (110× 10^−12^ - 0.86× 10^−5^) + MF (1.5 mM)	27.5 × 10^−8^	12.5	0.005
TAX (94× 10^−11^- 1.47× 10^−4^ M)	1.47 × 10^−4^	-	
TAX (94× 10^−11^- 1.47× 10^−4^ M) + MF (1.5 mM)	5.86 × 10^−6^	25	0.045
VBL (3.84× 10^−11^ - 0.03× 10^−4^ M)	0.11 × 10^−6^	-	
VBL (3.84× 10^−11^ - 0.03× 10^−4^ M) + MF (1.5 mM)	0.44 × 10^−8^	25	0.060
VCR (3.84× 10^−11^ - 0.03× 10^−4^ M)	0.2 × 10^−7^	-	
VCR (3.84× 10^−11^ - 0.03× 10^−4^ M) + MF (1.5 mM)	2.4 × 10^−9^	8.33	0.119
ETP (5.12× 10^−10^ - 0.04× 10^−3^M	0.18 × 10^−4^	-	
ETP (5.12× 10^−10^ - 0.04× 10^−3^M) + MF (1.5 mM)	0.51 × 10^−6^	35	0.003
ELP (2.56× 10^−10^ - 0.02× 10^−3^M)	0.02 × 10^−3^	-	
ELP (2.56× 10^−10^ - 0.02× 10^−3^M) + MF (1.5 mM)	0.32 × 10^−7^	625	0.001
HHG (25.6× 10^−12^ – 0.2× 10^−5^M)	0.6 × 10^−6^	-	
HHG (25.6× 10^−12^ – 0.2× 10^−5^M) + MF (1.5 mM)	2.25 × 10^−8^	27	0.012

^a^The data are based on the mean of absorbance measurements from three independent experiments

^b^Sensitization ration = IC_50_ of drug / IC_50_ of drug + MF

^c^ *P* Value for the combined treatment with drug and MF vs. drug alone

## Discussion

MF inhibited the proliferation of colorectal cancer cells. Both SW1116 and SW837 were significantly inhibited with IC_50_ 1.73 and 1.90 mM, respectively. Similar studies have been recently published [24] establishing a dose-dependent inhibition of colon cancer cells (HCT15 and HT-9) growth in the presence of *p*-coumaric acid, a congener of ferulic acid. Both HCT-15 and HT-29 cell growth was inhibited at IC_50_ 1.4 and 1.6 mM, respectively. These findings support our results for colorectal cancer cell lines SW1116 and SW837.

Janicke et al. [25] have reported that ferulic acid (FA) and para-coumaric acid (p-CA) dropped the cell count of colonic endothelial tumor cell line Caco-2 by 43-75 % compared with the control, after 2–3 days of treatment at 1.5 mM concentration. Recently, Eroĝlu et al. [26] have reported 0.3 mM and 0.5 mM half-maximal inhibitory concentration of ferulic acid for prostate cancer cell lines PC-3 and LNCaP, respectively. This shows that the effectual concentration of phenolic acids is tumor-type dependent and therapeutically relevant as chemo-adjuvant [27]. Thus, IC_50._ found in our study is within the published range. Studies reported by Raza et al. [28] indicated that methyl ferulate (MF), methyl p-coumarate (MpC), and pulegone 1,2-epoxide (PE) were non-toxic/non-irritant and may be useful for medicinal purposes [28].

In addition, the high bioavailability of 28–230 mg of *p*-coumaric acid, after consumption of 200 g plum, has been reported [27]. In a colonic volume of 200 mL, this would yield a concentration range 0.85–7.0 mM/L indicating that IC_50_ values, against colon or colorectal cancer cells are internally achievable.

Bioavailability not only varies amongst individuals but also depends upon other factors such as sex, age, ethnicity etc. making bioavailability studies difficult. The biggest snag is that the bioavailability from a mixture of phenolics is dependent on the type of food intake whereas in cell culture environment, it is not only singular but also constant [25].

One of the most important criteria anticancer drugs must meet is the selective killing of cancer cells with minimal damage to normal cells. MF is a common natural product in the diet, and is non-toxic to normal human fibroblasts (Fig. 1B). Many polyphenolic compounds that show growth inhibitory effect against different types of cancers have been reported [26, 28]. Inhibition of the colony formation is an important characteristic for many chemotherapeutic drugs, and MF showed a significant inhibition of colony formation, demonstrating the effectual anticancer potential of this molecule [29].

Growth and proliferation of cells are controlled by the cell cycle and its disruption causes an imbalance between proliferation and cell death, leading to cancer growth. Thus, anticancer agents, target the cell cycle to halt uncontrolled proliferation of cancer cells and initiate apoptosis [30]. Cell growth is controlled by several genetically defined checkpoints that ensure its coordinated progression through different stages of the cell cycle and monitor DNA integrity [31]. An analysis of the cell cycle after treatment with MF showed growth arrest in both SW1116 and SW837 cells lines at both the S and G_2_/M phases (Fig. 2), indicating the anticancer potential of MF. Many other natural phenolic acids have been reported to control the cancer cell cycle [32].

Our results support disruption of the cell cycle at the S phase, implying that MF interferes with DNA synthesis. It disrupts the progression of the cell cycle at the S- phase leading to apoptosis. Blocking damaged cells in the G_2_/M-phase provides ample time to repair DNA damage or permanently obstruct the damaged cells. Both of these responses are important in protecting organisms from tumor formation driven by an accumulation of mutations [33]. Many anticancer agents arrest the cell cycle at the G2/M phase and then induce apoptotic cell death [34]. G2/M phase cell cycle arrest involves targeting tubulin or disrupting the tubulin-microtubule equilibrium [35], which suggests that G2/M arrest by MF may play a role in the inhibition of microtubule dynamics.

One of the challenges faced in cancer treatment is that cancer possesses the ability to evade apoptosis, rendering cytotoxic drugs ineffective. The induction of apoptosis in tumor cells is considered expedient in the prevention of cancer [36]. A variety of natural products induce apoptosis in various tumor cells. Therefore, there is a good reason to search for apoptotic-inducing phytochemicals among both crude extracts and purified compounds [37]. In this study, MF showed a marked apoptotic effect on both the SW1116 and SW837 (Fig. 3) cell lines, indicating its therapeutic value as an anticancer agent. Many natural phenolic acids are apoptotic for different types of cancers [26, 29, 32].

The generation of ROS plays a vital role in cellular proliferation, differentiation and apoptosis. ROS stress is oncogenic and increases metabolic activity [38]. In the present study, ROS production after MF treatment was higher in MF-treated human colorectal cancer cells SW1116 (*P* ≤ 0.0001) and SW837 (*P* ≤ 0.008) than in untreated control cells (Fig. 4). Therefore, MF may be considered a potential exogenous ROS inducer for initiating apoptosis in human colorectal cancer cells. Our results are consistent with those reported for other phytochemicals targeting different types of cancers [19, 29]. ROS eliminate cancer cells by arresting the cell cycle at various checkpoints and therefore induce apoptosis [39].

The potential of MF to inhibit colorectal cancer cell invasion through a thin layer of extracellular matrix (ECM) was also investigated. It was found that MF greatly decreased the number of colorectal cancer cells SW1116 (*P* ≤ 0.006) (Fig. 5b, c) and SW837 (*P* ≤ 0.031) (Fig. 5e, f) at the bottom of the polycarbonate membrane compared with the number observed for the untreated control (Fig. 5a, d). It is known that phenolic compounds are effective against different types of tumors [26, 28, 40] which support our results.

NF-kB is a multi-subunit transcription factor which is maintained in the cytoplasm through interaction with the inhibitors of NF-kB. Upon dissociation, NF-kB moves into the nucleus and promotes cancer cell proliferation, angiogenesis and metastasis as well as inhibits apoptosis. Many different types of cancer, including colorectal cancer, show high NF-kB activity. In the current study, the DNA-binding activity of NF-kB in SW1116 (*P* ≤ 0.004) and SW837 cells (*P* ≤ 0.022) treated with MF was significantly reduced (Fig. 6A). Related phenolic acids, such as syringic acid methylester, gallic acid and caffeic acid phenylester, with similar effect, have been reported [41]. NF-kB activation transcriptionally activates several prosurvival genes including c-IAP1, c-IAP2 and XIAP [42]. A positive feedback loop, c-IAP2 and XIAP appear to trigger the activation of NF-kB [43]. Inhibition of NF-kB by MF would inhibit the prosurvival genes leading to an induction of apoptosis.

A recent approach in cancer therapy advocates the inhibition of the proteolytic activity of 26S proteasome [44]. Unlike normal cells, cancer cells increase proteasomal activity, which is essential for their survival and proliferation [44, 45]. Importantly, inhibitors of the 26S proteolytic unit of proteasome are known to induce apoptosis and cell cycle arrest only in neoplastic cells but not in normal cells [46]. Therefore, the proteasome has emerged as an attractive molecular target for cancer therapy [47]. We tested if the anticancer effect of MF was due its inhibitory potential for proteolytic activity of the 26S proteasome. We found that MF significantly inhibited the chymotrypsin-like activity (*P* ≤ 0.0001) (Fig. 6Ba) and the PGPH activity (*P* ≤ 0.0001) (Fig. 6Bb) but not the trypsin-like activity (*P* ≤ 0.447) (Fig. 6Bc) of the 26S proteasome in the cytosolic extract of SW1116. MF also significantly inhibited the chymotrypsin-like (*P* ≤ 0.0001) (Fig. 6Bd) and PGPH (*P* ≤ 0.0001) (Fig. 6Be) activities, as well as non-significantly affected the trypsin-like activity (*P* ≤ 0.065) (Fig. 6Bf), of 26S proteasome in the cytosolic extract of SW837. Our results are in agreement with those reported for other polyphenols in tumor cells [48]. The expression of two known substrates of proteasome, Bax and p27^kip1^, was also investigated. As expected, their expression markedly increased after treatment with MF, confirming MF’s potential to target the proteolytic activities of Ubiquitin Proteasome System (UPS).

Interestingly, it has shown that lactacystin and bortezomib enhance sensitivity of cancer cells that are resistant to routine chemotherapy [49]. Nevertheless, synthetic proteasome inhibitors have some toxicity. Therefore, proteasome inhibitors from natural food sources with minimal or no toxicity can be potential anticancer agents.

Cells can manage both endogenous and exogenous DNA damage through highly conservative DNA repair and cell cycle checkpoint signal pathways [50]. Several therapeutic agents can disrupt cell cycle regulation and impair checkpoint controls, inducing growth arrest and apoptosis in cancer cells [51]. Ample evidence shows that cyclin/ cyclin-dependent kinase (Cdk) complexes are modulated by cyclin-dependent kinase inhibitors (Cdkis), among which p21^WAF1/CIP1^ plays a major role in regulating the cell cycle at various checkpoints, leading to cell cycle arrest [52]. In the present study, MF differentially down-regulated the gene expression of Cdk1 and Cdk2. On the other hand, MF differentially up-regulated the gene expression of p19^INK4D^, p21^WAF1/CIP1^and p27^KIP1^ (Fig. 7a). Our results corroborate those reports for other phenolic acids [31, 32, 36]. With the identification of an increasing number of Cdks associated with cell cycle checkpoints, the identification of novel natural products, capable of selective inhibition of these kinases, offers a potentially attractive strategy for cancer therapy.

Based on the above mentioned results, it may be safely concluded that apoptosis may be involved in the inhibition of cell proliferation by MF. To investigate the mechanism underlying the apoptotic effect of MF, we assessed the expression of genes associated with apoptosis. MF differentially up-regulated the expression of the pro-apoptotic genes, including Bax, Bad, Bid, Bim, Apaf −1, Smac and caspases’ genes, including caspase-2, 3, 6, 7, 8 and 9 (Fig. 7b). At the same time, MF differentially down-regulated the anti-apoptotic genes, including cIAP-1, c-IAP-2, Bcl2, and FLIP (Fig. 7b). These results are in agreement with the reported data for other antitumor phenolic acids [26, 28]. Therefore, the increased expression of pro-apoptotic and decreased expression of anti-apoptotic genes indicates the apoptosis-inducing effect of MF on human colorectal cancer cells.

An exceptionally difficult problem in cancer treatment is multi-drug resistance, i.e., when cancer cells lose their sensitivity to multiple structurally different chemotherapeutics. This is one of the main problems in anticancer therapy [53]; hence, the search for phytochemicals that can sensitize cancer cells for chemotherapies is an important area of research. In this study, the rationale for combining MF with other standard treatment involving chemotherapeutic drugs was explored. Human colorectal cancer cells were exposed to two cytotoxic modalities that may act otherwise on molecular pathways, leading to synergistic/additive cancer cell death [54]. Our results indicate that MF differentially increases the sensitivity of colorectal cancer cells to standard chemotherapeutic drugs. Thus, MF markedly increased the sensitivity of SW1116 colon cancer cells to CPT (SR = 95), 5FU (SR = 1051), DOX (SR = 125), VIN (SR = 254), ETP (SR = 2204), ELP (SR = 4615), AMS (SR = 650) (Fig. 8, Table 2). On the other hand, MF enhanced the sensitivity of SW837 rectum cancer cells to 5FU (SR = 269) and ELP (SR = 625) (Fig. 9, Table 2). These results are in line with those reported in a recent study in which ferulic acid combined with aspirin demonstrated chemopreventive potential towards pancreatic cancer [55]. This study shows an excellent potential of small and simple molecules such as MF good anticancer activity. Numerous anticancer molecules from marine sources have identified but they all have very complex structural configuration making it hard to synthesize them. Similarly, other phytochemical like taxol and vinca alkaloids are very complex in nature. Besides, they have other undesirable effects on human health. MF is a simple universally present nontoxic phenolic molecule with excellent potential that can be used either singly or in combination therapy for colorectal cancer. Further studies must be carried out to explore additional potentialities of MF.

## Conclusion

MF was isolated and identified for the first time from *Tamarix aucheriana*, MF showed a multifaceted anti-proliferative effect through cell cycle arrest, induction of apoptosis, ROS generation, inhibition of cell invasion, NF-kB DNA-binding activity and various proteolytic activities of proteasome. Furthermore, MF up-regulated the expression of pro-apoptotic and Cdkis genes. In contrast, MF down-regulated the expression of anti-apoptotic and Cdk genes. Although additional investigations of other cell lines and in vivo animal models are required to strengthen these findings, this study highlights the potential of MF, a common dietary phenolic molecule that may be valuable for the pharmaceutical industry.

## Abbreviations

^13^C-NMR: Carbon-13 nuclear magnetic resonance
5FU: 5-fluorouracil
AMC: 7-amido-4-methyl-coumarin
AMS: Amsacrine
APD: Aphidicolin
ATCC: American Type Culture Collection
Cdkis: Cyclin-dependent kinase inhibitors
Cdks: Cyclin-dependent kinases
CO_2_: Carbon dioxide
CPT: Camptothecin
CRC: Colorectal cancer
d: Day
DCF-DA: Dichlorofluorescein diacetate
DMSO: Dimethylsulfoxide
DOX: Doxorubicin
ECM: Extracellular matrix
EIA: Enzyme immunoassay
ELISA: Enzyme linked immunosorbent assay
ELP: ellipticine
EMEM: Eagle’s Minimum Essential Medium
ETP: Etoposide
FBS: Fetal bovine serum
FITC: Fluorescein isothiocyanate
h: Hour
H^1^-NMR: Proton nuclear magnetic resonance
HHG: Homoharrigtonine
HRP: Horse-reddish peroxidase
IC_50_: The half maximal inhibitory concentration
IR: Infrared
MAPK: Mitogen-activated protein kinases
MF: Methylferulate
MS: Mass Spectrometry
MTT: 3-(4,5-dimethylthiazol-2-yl)-2,5-diphenyltetrazolium bromide
NFkB: Nuclear factor kappa B
OD_t_: Optical density of treated
OD_ut_: Optical density of untreated
OXP: Oxaliplatin
PBS: Phosphate buffered saline
PGPH: Peptidyl-Glutamyl Peptide-Hydrolyzing
PI: Propidium iodide
Real time qPCR: Real time quantitative polymerase chain reaction: qPCR
R_F_: Retardation factor
RNase: Ribonuclease
ROS: Reactive oxygen species
RT- PCR: Reverse transcriptase PCR
SR: Sensitization Ratio
TAX: Taxol
TLC: Thin layer chromatography
UV: Ultraviolet
UPS: Ubiquitin Proteasome System
UT: Untreated
VBL: Vinblastine
VCR: Vincristine
